# Integrated analysis of transcriptome and milk metagenome in subclinical mastitic and healthy cows

**DOI:** 10.5713/ab.21.0495

**Published:** 2022-01-21

**Authors:** Jinning Zhang, Xueqin Liu, Tahir Usman, Yongjie Tang, Siyuan Mi, Wenlong Li, Mengyou Yang, Ying Yu

**Affiliations:** 1 Key Laboratory of Animal Genetics, Breeding and Reproduction, Ministry of Agriculture and National Engineering Laboratory for Animal Breeding, College of Animal Science and Technology, China Agricultural University, Beijing, 100193 China; 2 College of Veterinary Sciences and Animal Husbandry, Abdul Wali Khan University, Mardan, 23200, Pakistan

**Keywords:** Host-microbe Interplay, Metagenome, Subclinical Mastitis, Transcriptome

## Abstract

**Objective:**

Abnormally increased somatic cell counts (SCCs) in milk is usually a sign of bovine subclinical mastitis. Mutual interaction between the host and its associated microbiota plays an important role in developing such diseases. The main objective of this study was to explore the difference between cows with elevated SCCs and healthy cattle from the perspective of host-microbe interplay.

**Methods:**

A total of 31 milk samples and 23 bovine peripheral blood samples were collected from Holstein dairy cattle to conduct an integrated analysis of transcriptomic and metagenomics.

**Results:**

The results showed that *Ralstonia* and *Sphingomonas* were enriched in cows with subclinical mastitis. The relative abundance of the two bacteria was positively correlated with the expression level of bovine transcobalamin 1 and uridine phosphorylase 1 encoding gene. Moreover, functional analysis revealed a distinct alternation in some important microbial biological processes.

**Conclusion:**

These results reveal the relative abundance of *Ralstonia* and *Sphingomonas* other than common mastitis-causing pathogens varied from healthy cows to those with subclinical mastitis and might be associated with elevated SCCs. Potential association was observed between bovine milk microbiota composition and the transcriptional pattern of some genes, thus providing new insights to understand homeostasis of bovine udder.

## INTRODUCTION

Bovine subclinical mastitis, characterized by elevated milk somatic cell counts (SCC), is not only associated with dairy animals’ health and welfare, but also with the quality of dairy products. Amongst numerous factors, bacterial infection is one of the major causes of subclinical mastitis. Under normal circumstances, the composition of microbial community is in dynamic balance in the mammary gland of lactating cows, however, various endogenous and exogenous upsets can disrupt intramammary homeostasis, resulting in dysbiosis in the mammary gland, thus, leads to mastitis or subclinical mastitis. The host-associated factors and microbial profile are main determinants of the bovine udder homeostasis [1,2].

Recently, multiple advances have been gained in studies of milk microbiota and its association with the bovine mammary health status. Historically, bacteria detected in milk was linked to external contamination but latest researches suggested otherwise [3]. Isolation and cultivation of milk bacteria has yielded a lot of results in common clinical or subclinical mastitis pathogens identification. Various metagenomics studies have shown that milk microbiota composition can reflect the mammary gland health state to greater extent. A shift in microbiota composition is reported in milk from subclinical mastitis cows [1,4–6], however, there lacks a consensus on which microbiota constitutes the microbial community of milk from healthy quarters.

Host-microbe interplay, shaped by host defense against potential harmful microorganisms and the response of microorganisms to the host immune system attack, is present in many immune-mediated diseases including bovine mastitis and subclinical mastitis [2]. Alternation in host gene expression pattern of specific pathogen-induced subclinical mastitis has also been well studied in recent years [7]. Despite lots of bacterial infection experiments performed *in vitro* to elucidate host-bacteria interaction in bovine mammary diseases, the mechanism of the interplay between host and colonized microbiota as a whole remains vague. To our knowledge, a combined analysis of milk microbiota and host gene expression in attempt to unveil the relationship among host immune response, microbiota and bovine subclinical mastitis has not been reported till date.

The present study offers a new perspective to understand how microbes interact with the host in bovine subclinical mastitis and to elucidate the role of the gene-bacteria interplay in developing such diseases. We implemented a comparative analysis based on the host transcriptome and metagenome sequencing and investigated a differential gene expression profile between subclinical mastitis and healthy cows.

## MATERIALS AND METHODS

### Sample and phenotype collection

Procedures for collection of samples were approved by the Animal Welfare Committee of China Agricultural University, Beijing, China and were conducted strictly following the regulations and guidelines established by this committee (approval number: AW10111202-1-3). All cows sampled in this research were guaranteed to have no records of clinical mastitis for three consecutive months, and were reared in the same dairy farm in Beijing, Northern China. Milk samples collection was performed after a regular disinfection procedure i.e. teat cleaning with 70% ethanol and the first three streams of milk were discarded [8]. Milk from each of the four quarters was collected in a 50 mL-sterile falcon tube, evenly mixed together and kept on dry ice during the whole collection process. For each cow, two milk samples were taken with one put in −80°C for later DNA extraction and the other sent to Beijing Dairy Cattle Center for SCC measurement. Other phenotype information was acquired according to records provided by the dairy farm. A total of 23 peripheral blood samples were aseptically taken from caudal vein and stored in 10-mL pre-added ethylenediaminetetra-acetic acid-K2 vacutainers on the same day the milk samples were collected.

### DNA, RNA extraction and sequencing

Total genomic DNA was extracted from milk using cetyltrimethyl ammonium bromide. The integrity and purity of DNA was tested using 1% agarose gel electrophoresis. Total RNA was isolated from peripheral white blood cells using Trizol reagent (Ambion, Waltham, MA, USA) following the manufacturer’s instructions. Purity of RNA was measured by Nanodrop (Thermo Fish, Waltham, MA, USA) and RNA integrity was assessed using electrophoresis and Aglient 2100 BioAnalyzer. Qubit 2.0 Fluorimeter was used for accurate quantification of DNA and RNA. Both metagenomic shotgun sequencing and RNA-seq were performed on Illumina NovaSeq 6000 platform (Illumina, San Diego, CA, USA), preceded by a construction of 150-bp paired-end library. The average percent of bases scoring Q30 or above were over 90% for reads generated from either metagenomic shotgun sequencing or RNA-seq techniques.

### Metagenomics analysis

Based on a threshold of 100,000 SCC/mL, the samples were divided into MS (medium SCC) and LS (low SCC) group [8]. Low quality reads, adaptors and host reads filtering were accomplished using KneadData. The quality of all clean data was assessed utilizing FastQC first before subsequent analyses. Non-host reads that passed quality control were taken for taxonomic classification using Kraken2 and abundance re-estimation using Bracken (v 2.6). Eukaryotes and viruses were discarded from the analysis. *De novo* assembly of reads into contigs was conducted by Megahit (v 1.2.8) and protein-coding gene prediction was performed using Prodigal (v 2.6.3). Redundant sequences of coding sequence predicted were then removed by CD-HIT (v 4.8.1) in order to facilitate downstream analysis. The remaining non-redundant representative sequences then served as an index for Salmon alignment-free quantification and also the input to eggnog-mapper (v 2.0.1) for functional annotation. Alpha diversity indices (Shannon, Chao1, Simpson) at different sampling depths were calculated using function diversity using R package vegan. Difference of alpha diversity between the two groups was examined using Wilcoxon rank-sum test. Bray-Curtis distance matrices based on relative abundance table at different taxonomic levels were built up using function *vegdist* in vegan and permutational multivariate analysis of variance was done by *adonis* to examine the effect of various experimental factors (SCC, parity, lactation stage) on differences among samples (Bray-Curtis distance ~ SCC + parity + lactation stage). Dissimilarity among samples (beta-diversity) was visualized with function *pcoa* in ape package. To eliminate the effect of library size, edgeR was utilized to determine differentially abundant taxa (DAT) and metagenomic function.

### Transcriptomic analysis

Raw reads generated from RNA-seq were subjected to strict quality control (Trimmomatic v0.39) and were then aligned to *Bos taurus* ARS-UCD1.2 using STAR (v 2.7.9a) with an average mapping rate over 90%. Reads mapped to the reference genome were quantified by feature Counts in subread (v 2.0.2) package followed by TPM normalization using StringTie (v 2.1.5). Subsequent analyses were carried out using R package. Differentially expressed genes (DEG) were identified by edgeR. Gene ontology (GO) and Kyoto encyclopedia of genes and genomes (KEGG) pathway enrichment analysis were conducted using online website KOBAS (kobas.cbi.pku.edu.cn/kobas3).

### Integrated analysis of microbiome and host gene expression

To find the correlation between co-expression gene modules and DAT, weighted correlation network analysis and module-trait relationship assessment were implemented by WGCNA using top 5,000 genes with the largest median absolute deviation (MAD). Pearson correlation coefficients of the relative abundance of DAT and genes both identified as DEGs and found in modules associated with DAT were calculated using function *cor.test* in R.

## RESULTS

### Brief summary of phenotypes

A total of 31 (MS = 13, LS = 18) milk samples were obtained and used for sole metagenomics analysis. The parity of each selected cow was less than three and days in lactation ranged from 54 to 164 d (97±30.5 d). Distribution of SCC, parity and lactation are presented in Supplementary Figure S1. In general, 23 (MS = 8, LS = 15) samples that had both transcriptome data and metagenome data were contained in integrative analysis.

### Overview of taxonomic profiling in samples

**Figure 1:**
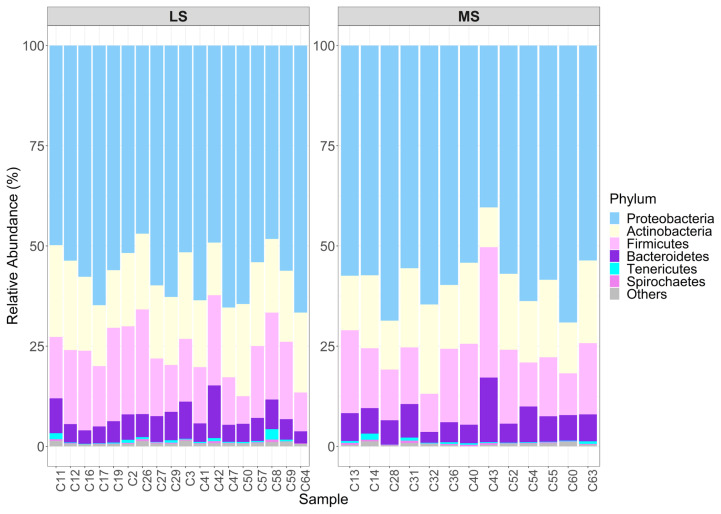
Relative abundance of top six highly representative bacteria at phylum level within each sample. MS, medium somatic cell count; LS, low somatic cell count.

A total of 35 phyla were observed, of which 4 phyla (*Proteobacteria* 54.73±14.35; *Actinobacteria* 16.47±6.56; *Firmicutes* 19.71±12.83; and *Bacteroidetes* 9.40±6.70) were shared across all samples. Top 6 most abundant phyla selected based on the average relative abundance were *Proteobacteria*, *Actinobacteria*, *Firmicutes*, *Bacteroidetes*, *Tenericutes*, and *Spirochaetes* (Figure 1). Additionally, 70 classes, 166 orders, 375 families and 1,413 genera were identified. Species level were excluded from subsequent analyses due to inadequate sequencing depth for accurate classification at this level.

### Comparison of diversity and composition of microbial communities

**Figure 2:**
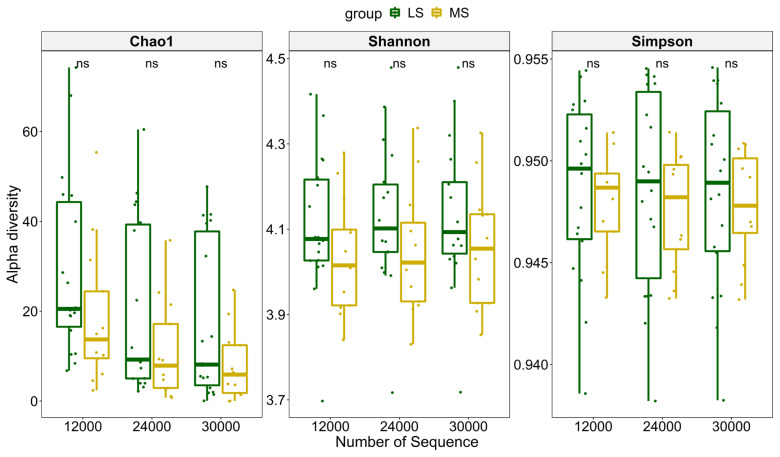
Comparison of microbial diversity between samples in MS and LS group at different sampling depths. Chao1, Shannon, Simpson are three different diversity indices. ns, means that the difference is not significant (p>0.05). MS, medium somatic cell count; LS, low somatic cell count.

To compare richness of the microbial community in the two groups, three different indices were used to measure the overall diversity in each sample (Figure 2). The significance was calculated using paired Wilcoxon rank-sum test. The results showed that samples, subsampled at a depth of 12,000, 24,000, and 30,000 had no significant differences between MS and LS group using any of the three alpha diversity indices, indicating the richness difference was quite subtle. We further explored the impact of SCC, parity and lactation stage on dissimilarities of microbial composition among samples using PERMANOVA. The result suggested that SCC has little impact on Bray-Curtis dissimilarity at almost all classification levels (p>0.05). Moreover, parity and lactation stage also accounted for little of the variation on overall microbial composition (Table 1). Principal coordinate analysis also showed no clustering tendency within either group and that the discrepancy between MS and LS samples remained unclear at any classification level (Supplementary Figure S2).

### Differentially abundant taxa identification

**Figure 3:**
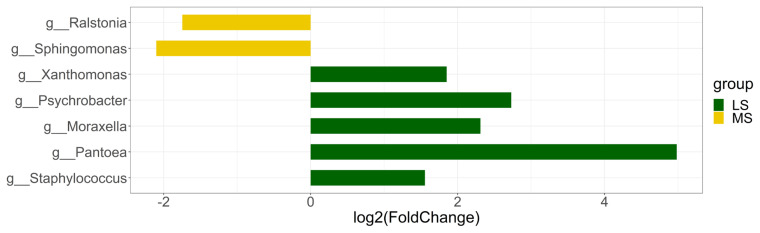
Genus with significant differences between MS and LS (LS vs MS). Genus enriched in MS and LS are shown in yellow bars and green bars, respectively. MS, medium somatic cell count; LS, low somatic cell count.

To evaluate the difference of relative abundance of specific taxon between the two groups, we utilized edgeR to normalize the counts and then identify SCC-associated taxa. Genus level was mainly focused because the difference in other lower-ranked classification levels was already captured at genus level. To further avoid the bias caused by sequencing depth, only taxa existent in at least 30% of samples in either group were kept for differential abundance analysis. Differentially abundant taxa were selected based on |log2 (fold change)| ≥1 and p<0.05 (Figure 3). A total of 7 genera were identified using this threshold. Two Gram-negative bacteria - *Ralsotina* and *Sphingomonas* were enriched in MS group while the abundance of *Xanthomonas*, *Psychrobacter*, *Moraxella*, *Pantoea*, *Staphylococcus* was significantly higher in LS group.

### Identification of genes both related to somatic cell counts and differentially abundant taxa

In order to find out whether certain host genes could potentially get involved in the interplay between bacteria and host, a combined analysis of DEG and weighted gene co-expression network was carried out to evaluate the correlation between host phenotype (SCC), gene expression pattern and the relative abundance of DAT. We first performed a DEGs analysis in MS and LS group. DEGs were identified with |log2 (fold change)| ≥1 and p<0.05. In general, 355 genes were differentially expressed in the two groups, of which 297 genes were up-regulated and 58 were down-regulated in MS compared with LS. Next we ranked genes according to MAD and the top 5000 genes were selected to be clustered into modules by WGCNA based on the correlation.

**Figure 4:**
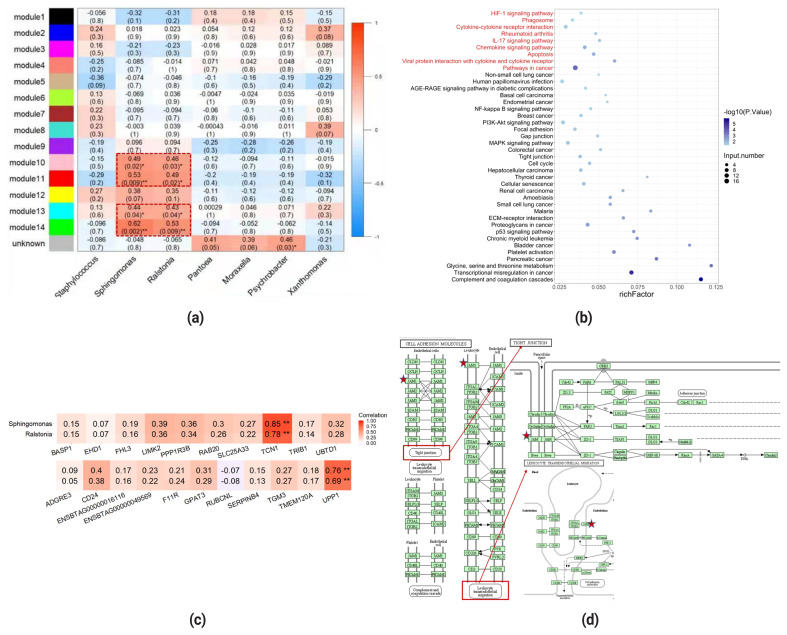
Overlap of genes correlated with differentially abundant taxa and genes differentially expressed in MS and LS. (a) Heatmap of module-trait relationship. Each row corresponds to eigengene of a module and column to the relative abundance of a differentially abundant taxa. (b) KEGG pathway enrichment results for DEGs. Pathways labeled in red represent pathways enriched by both DEGs and genes in M11. Rich factor was calculated based on the number of DEGs enriched in this pathway and the number of background genes. (c) Correlation between genes shared by both DEGs and M11 and the relative abundance of *Ralstonia* and *Sphingomonas*. Significance was established at p<0.05). ** Stands for p<0.01. (d) Map of the KEGG signaling pathways of tight junction and leukocyte transendothelial migration. Protein transcribed from *F11R* was marked with red asterisks. MS, medium somatic cell count; LS, low somatic cell count; KEGG, Kyoto encyclopedia of genes and genomes; DEG, differentially expressed genes.

To further explore which module was potentially related to DAT, relative abundance of each seven differentially abundant genera identified above was used as a trait and the correlation between it and each module eigengene was evaluated. Four (M10, M11, M13, M14) out of 14 modules exhibited significant relevance to the relative abundance of *Ralsotina* and *Sphingomonas*, which were enriched in MS group (Figure 4a). A total of 297 up-regulated DEGs were mapped to genes in these four modules to find genes that were both related to SCC and MS-enriched bacteria. 0, 21, 2, 2 genes in M10, M11, M13, M14 overlapped with those in DEGs, respectively (Supplementary Figure S3). Relatively high overlap between genes in M11 and DEGs provided evidence that genes in M11 might play a role in host-microbiota interaction.

To get functional interpretation of these genes and pathways they were involved in, we conducted GO and KEGG pathways enrichment analysis. Because of the inadequate number of overlapping genes to complete enrichment analyses, an overlap between GO and KEGG pathways enriched by genes in M11 and DEGs were investigated instead. As shown in Figure 4b, approximately nine KEGG pathways were shared by both sets. Several pathways related to inflammation and immune response were identified, such as phagosome, cytokine-cytokine receptor interaction, interleukin-17 (IL-17) signaling pathway, chemokine signaling pathway, apoptosis. The overlapping GO terms that fell in biological process categorical included chemokine-mediated signaling pathway, antimicrobial humoral immune response, neutrophil chemotaxis, which were also associated with immune signals, suggesting that there was an increase in the immune system activities in MS group compared with LS group and were consistent with the physiological process during subclinical mastitis, because subclinical mastitis was usually characterized by active immune responses (Supplementary Figure S4). Equally important is that the result also demonstrated a higher level of kinase and phosphatases activities in MS which was typically involved in initiation of immune cells activation in response to antigens. Despite the fact that none of the 21 genes shared by DEGs and M11 was enriched in these pathways or terms, the result infers that the intensity of immune responses varied from subclinical mastitis cows to healthy cows, and also from cows with higher abundance of *Ralstonia* and *Sphingomonas* to cows with lower abundance of those, though the variation might not be obvious. However, one gene, *F11R* was found to be involved in two different enriched KEGG pathways. *F11R* was significantly enriched in tight junction using DEGs for pathway enrichment analysis and leukocyte transendothelial migration using M11 genes, indicating its role in initiation of inflammatory immune response and barrier function maintenance. To further determine a relevance between the change in expression level of some specific genes and the relative abundance of *Ralstonia* and *Sphingomonas*, Pearson correlation was used to find genes directly linked to these two kinds of bacteria (Figure 4d). The results of correlation test showed that transcobalamin 1 (*TCN1*) and uridine phosphorylase 1 (*UPP1*) had a strong correlation with either *Ralstonia* (0.78, 0.69, respectively) or *Sphingomonas* (0.85, 0.76, respectively).

### Annotation of metagenomic biological function potentially contributing to increase in somatic cell count

**Figure 5:**
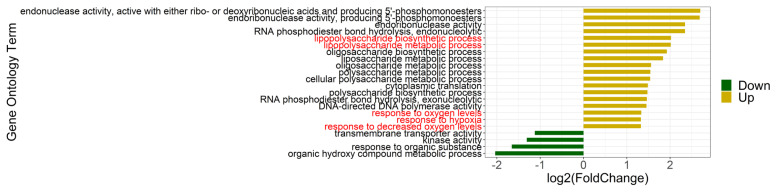
Differentially enriched GO terms (MS vs LS). Significance was established using p<0.05 and |log2(fold change)| ≥1. Yellow bars indicate enriched GO terms in MS and green bars indicate enriched GO terms in LS. MS, medium somatic cell count; LS, low somatic cell count; GO, gene ontology.

Based on the functional annotation of metagenome, we sought to detect the biological processes that either intensified or weakened in MS group. Differentially enriched GO terms determined by edgeR are shown in Figure 5, of which four were less abundant in MS compared with LS, and 18 showed the opposite tendency. The four down-regulated GO in MS were organic hydroxy compound metabolic process, transmembrane transporter activity, kinase activity and response to organic substance. Notably, lipopolysaccharide (LPS) biosynthetic and metabolic process, response to oxygen levels and hypoxia were up-regulated. It is noteworthy that the activities of some enzymes involved in DNA replication and nucleotide synthesis also increased in MS, suggesting active bacterial reproduction was underway.

## DISCUSSION

Though, previous studies have identified lots of candidate genes and potential microbial biomarkers associated with subclinical mastitis, however, to our knowledge this is the first study to combine bovine transcriptome and milk metagenome to explore the connections of host and microbiota in dairy cattle subclinical mastitis.

The present study identified a difference in the relative abundance of *Ralstonia* and *Sphingomonas* between MS and LS group. Previous studies reported association of *Ralstonia* with mastitis and subacute mastitis in human [9] and detected both *Ralstonia* and *Sphingomonas* as main components of donkey milk [10,11]. *Ralstonia* is well known to come from environmental resources and was reported to be associated with water contamination and it shows greater adaptability to adverse conditions than many other bacteria [4,12]. Same as *Ralstonia*, *Sphingomonas* are also environmental bacteria and can be found in tap water or soil. Notably, two species belonging to *Sphingomonas* genus (*S. paucimobilis* and *S. maltophilia*) were isolated from milk samples of clinical mastitis cows [13]. Another milk microbiota profiling study reported that *Sphingomonas* was more enriched in culture negative clinical milk samples, whereas, *Ralstonia* was more easily detected in the microbiota of healthy quarters [4]. Though, no direct association was identified between the presence of the two bacteria and clinical or subclinical mastitis, however, these were reported to be related with some human diseases, such as colitis associated cancer [14], cystic fibrosis [15] and gastric inflammation [16]. Besides, opportunistic infection can sometimes be caused by non-pathogenic bacteria.

Common KEGG pathways and GO enriched by genes in modules highly correlated with *Ralstonia* and *Sphingomonas* and genes in DEGs were associated with immune responses. Apart from that, a significantly positive correlation between the two bacteria and expression level of *TCN1* and *UPP1* was determined in this study (p<0.05). *TCN1* encodes cobalamin (a vitamin B12-binding protein) and *UPP1* is the coding gene of uridine phosphorylase 1. High expression of *TCN1* and *UPP1* was present in many malignant cancers. *TCN1* was associated with breast phyllodes tumors [17] and was noticed to be a negative indicator in prognostic evaluation of rectal cancer [18]. High expression of *UPP1* was reported in breast cancer and thyroid cancer cells [19]. Keeping in view the combined role of cobalamin and UPP1 in cell metabolism, the up-regulation of *TCN1* and *UPP1* in MS was reasonable, due to its involvement in a wide range of basic biological processes.

Metagenomic functional annotation results further demonstrated active host-microbe interplay in MS group. For Gram-negative bacteria, LPS is an important component of outer membrane and plays an essential part in host-pathogen interaction during infection [20]. Similarly, molecular oxygen exhaustion has been proved to be in relevance to some pathological states, such as inflammation and bacterial infection. This was also consistent with the result that hypoxia inducible factor (HIF-1) signaling pathway was significantly enriched by host genes in M11 and DEGs. The findings of the present study are in line with the previous study showing that the therapeutic effect of alpha-linolenic acid based intra-mammary nano-suspension on LPS induced mastitis in rat resulted in suppression of the HIF-1α [21]. Contrary to previous studies [5,6], our results showed that no distinct differences of global diversity and richness existed between the microbial communities in subclinical mastitis cows and in healthy ones due to great variation within group. However, we can see most samples in LS had a higher index, implying greater microbiota diversity in healthy milk samples. Similar finding in beta-diversity pattern further illustrated that there was no clear separation in overall microbial composition in different groups. Besides, sample distance – variable association assessed by PERMANOVA demonstrated that none of the three variables (SCC, parity, and lactation) can perfectly explain the dissimilarity. This was probably because the SCC of cows we sampled was relatively concentrated and phenotype such as parity or lactation stage is unlikely to have a huge impact on composition of microbial community since it is usually in a dynamic balance [22]. Another important reason that may account for this phenomenon is the relatively inadequate depth of our metagenome sequencing to cover some rare bacteria species, as microbiota richness is sensitive to perturbation with rare bacteria [23].

In this study, metagenome sequencing was implemented instead of 16S rRNA gene sequencing, due to its advantage in gene function annotation. However, host contamination caused by somatic cells in milk posed a huge problem for this study and led to inadequate sequencing depth for rare species identification and other functional analyses i.e. virulence factors abundance investigation. However, to maintain statistic power, comparison between groups was all based on relative abundance, a typical library size correction method in metagenomics analysis. And we set a high threshold for the minimum sample size of each group that specific bacteria or GO term needed to be present in order to be kept for subsequent comparative analysis.

Transcriptome analysis was performed based on RNA derived from peripheral blood instead of milk mainly because of an ease of RNA extraction and representativeness of peripheral blood transcriptomic signature [24]. We noticed that using 100,000 SCC/mL as a cut-off value for differentiation between healthy and subclinical mastitis is controversial in different countries [25]. Though, some studies reported that a history of bovine mastitis may also lead to changes in microbial community [26,27], however, we did not take the information into account at the beginning of this study. It is suggested to use strict criteria for selection of samples with different cut-off values and a higher sequencing depth in future study.

## CONCLUSION

In conclusion, we conducted an integrated analysis of milk microbiota and peripheral blood transcriptome profiling in healthy and subclincal mastitis cows in the current study. Although the exact mechanism behind the host-microbe interaction in the development of bovine subclinical mastitis has not been fully elaborated by us, our results highlight that identifying some specific host genes expression pattern changes may aid in detecting the infection in the bovine udder.

## Figures and Tables

**Table 1:** Assessment of variation explained by various experimental factors at different classification level using PERMANOVA

Classification level	SCC group	Parity	Lactation stage
Phylum	0.02 (0.48)	0.01 (0.80)	0.08 (0.10)
Class	0.03 (0.55)	0.06 (0.09)	0.05 (0.23)
Order	0.03 (0.66)	0.06 (0.06)	0.04 (0.34)
Family	0.03 (0.57)	0.05 (0.14)	0.03 (0.41)
Genus	0.05 (0.09)	0.05 (0.10)	0.03 (0.41)

SCCs, somatic cell counts.

R squared and significance are shown in the Table (Significance was established at p<0.05).

## SUPPLEMENTARY MATERIAL

**Supplementary Figure S1**. Distribution of parity, lactation days and SCC in all 31 cows. **Supplementary Figure S2**. Results of Principal coordinates analysis showing the dissimilarity between samples at different classification levels. **Supplementary Figure S3**. Differentially expressed genes (MS vs LS). **Supplementary Figure S4**. Gene Ontology (GO) enrichment results for DEGs.
