# Supplementary material for: Integrated analysis of transcriptome and milk metagenome in subclinical mastitic and healthy cows
Source: Anim Biosci. 2022 Jan 21;37(4):709–17. doi: 10.5713/ab.21.0495 (PMC10915226; doi:10.5713/ab.21.0495)
Supplement: Supplementary file 1 [file ab-21-0495-Supplementary-Figs.pdf]

Integrative analysis of transcriptome and bovine milk metagenome in subclinical mastitic and healthy cows

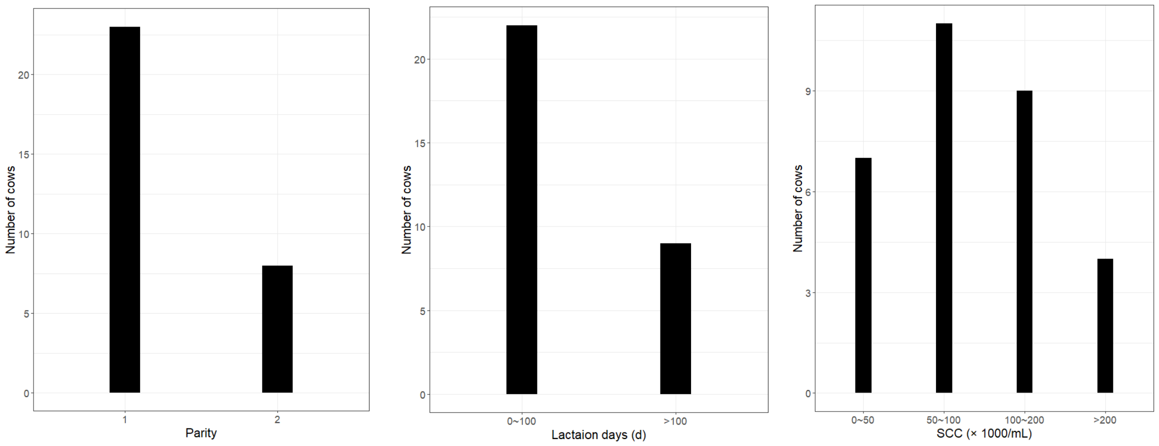

Supplementary Fig.1. Distribution of parity, lactation days and SCC in all 31 cows

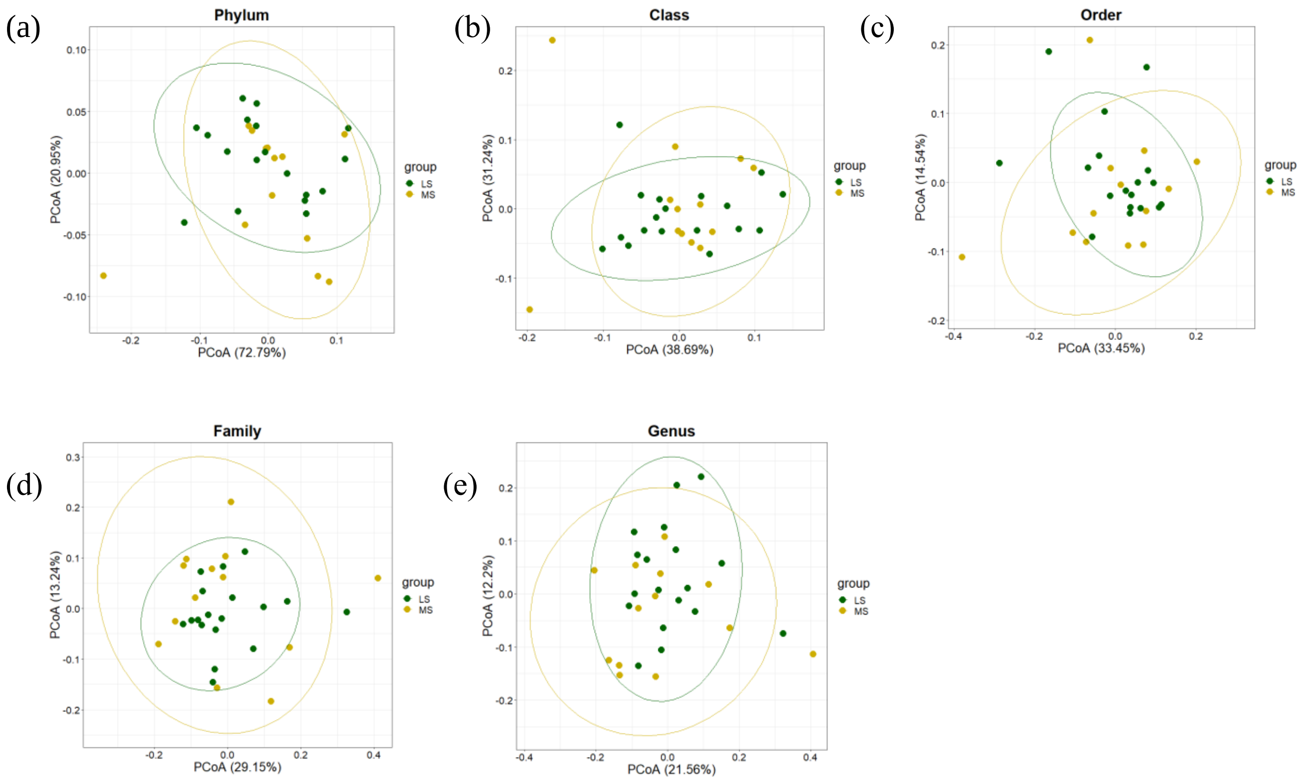

Supplementary Fig.2. Results of Principal coordinates analysis showing the dissimilarity between samples at different classification levels. (a) Phylum level (b) Class level (c) Order level (d) family level (e) genus level

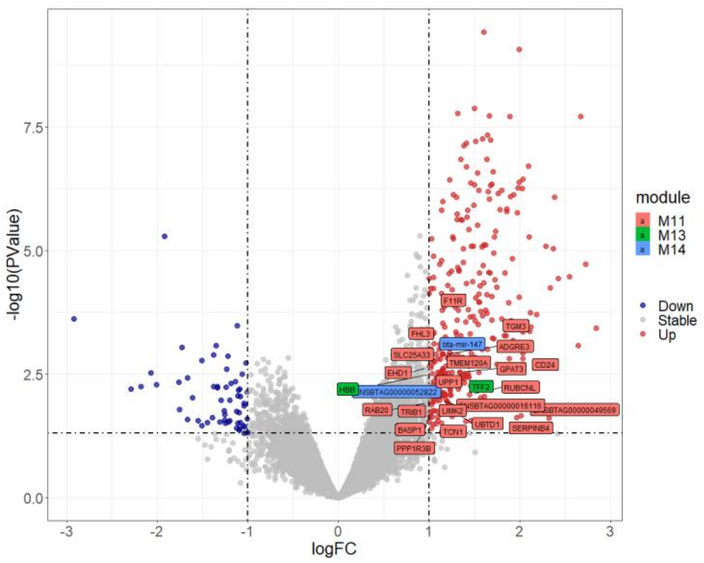

Supplementary Fig.3. Differentially expressed genes (MS *vs* LS**).** Genes shared by DEGs and modules associated with MS-enriched bacteria are labeled in this figure.

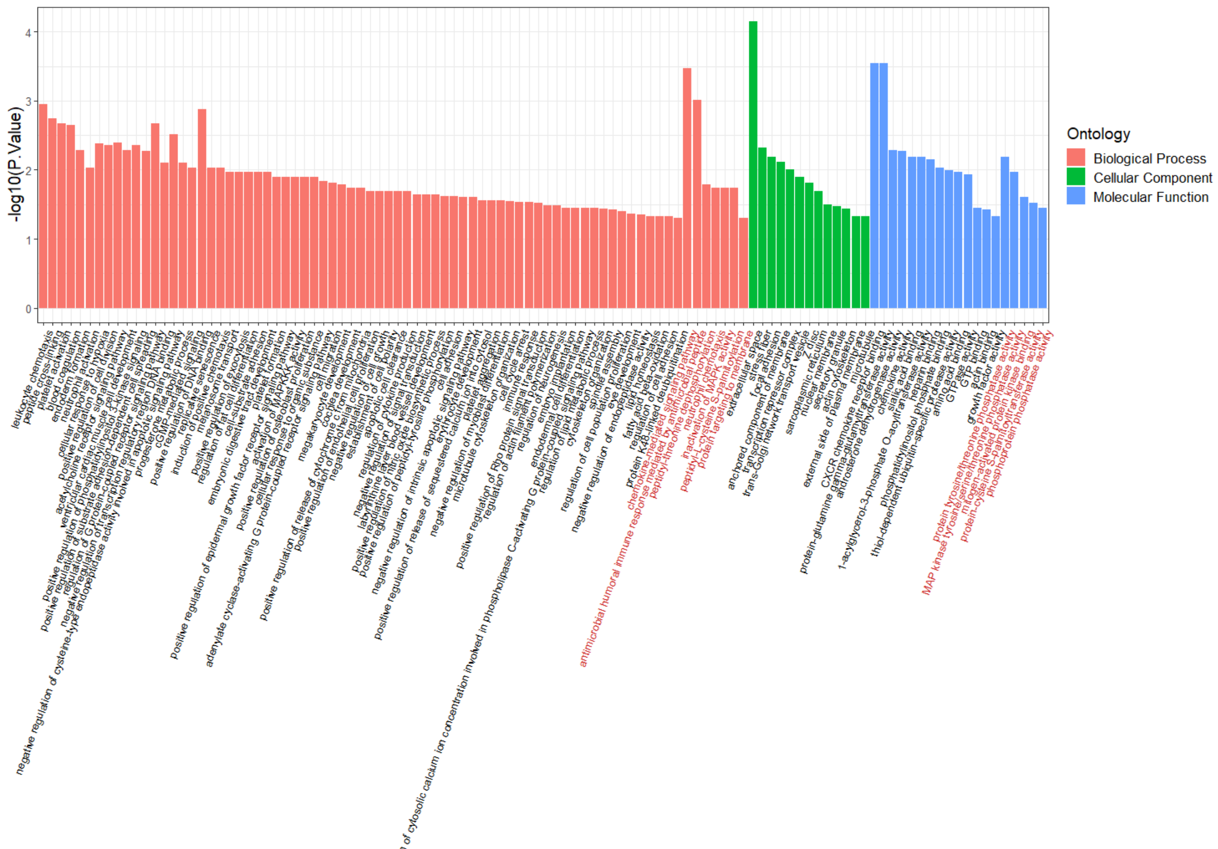

Supplementary Fig.4. Gene Ontology (GO) enrichment results for DEGs. Pathways labeled in red represent pathways enriched by both DEGs and genes in M11.
